# Supplementary material for: Ethyl Acetate Fraction of Amomum xanthioides Ameliorates Nonalcoholic Fatty Liver Disease in a High-Fat Diet Mouse Model
Source: Nutrients. 2020 Aug 13;12(8):2433. doi: 10.3390/nu12082433 (PMC7468949; doi:10.3390/nu12082433)
Supplement: Supplementary file 1 [file nutrients-12-02433-s001.zip › 20200721 Supplementary figure_1.pdf]

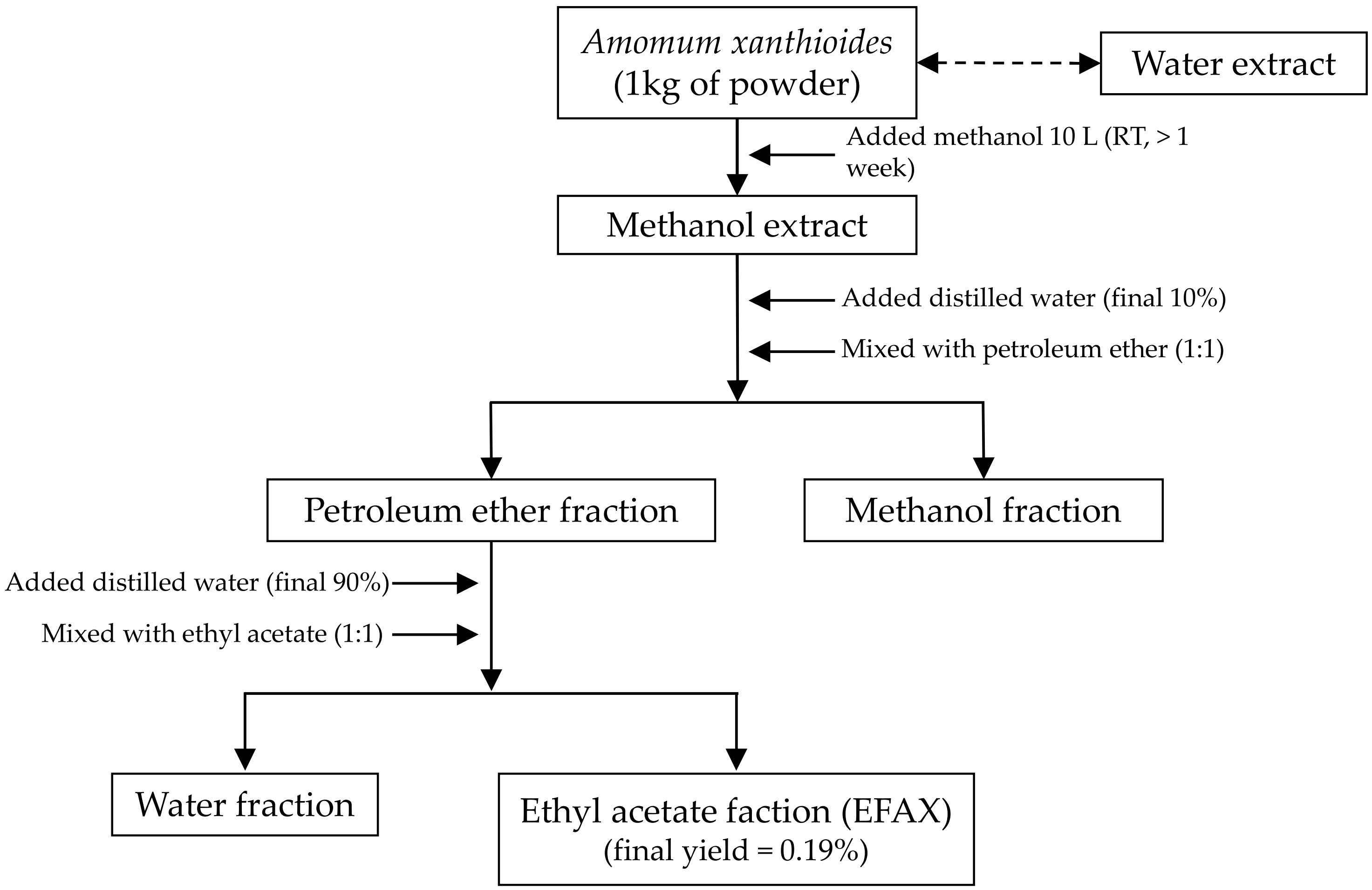

Supplementary figure 1. Flow chart of the ethyl acetate fraction of *Amomum xanthoides* (EFAX) acquisition
