# Supplementary material for: Ethyl Acetate Fraction of Amomum xanthioides Ameliorates Nonalcoholic Fatty Liver Disease in a High-Fat Diet Mouse Model
Source: Nutrients. 2020 Aug 13;12(8):2433. doi: 10.3390/nu12082433 (PMC7468949; doi:10.3390/nu12082433)
Supplement: Supplementary file 1 [file nutrients-12-02433-s001.zip › 20200721 Supplementary figure_2.pdf]

A

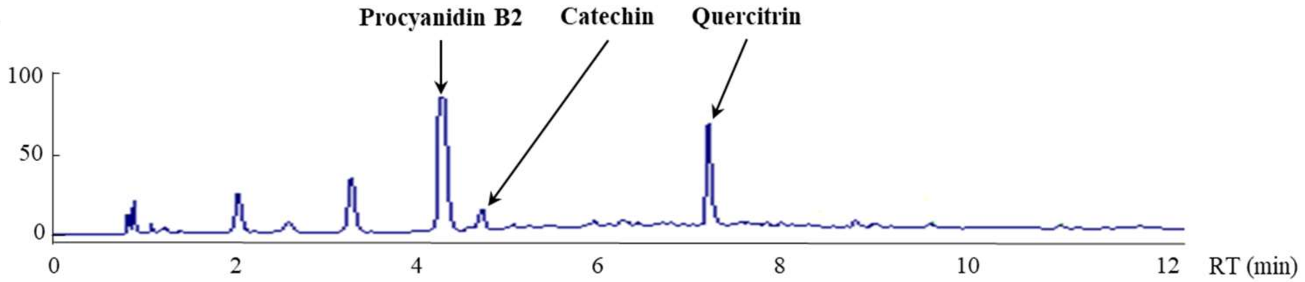

B

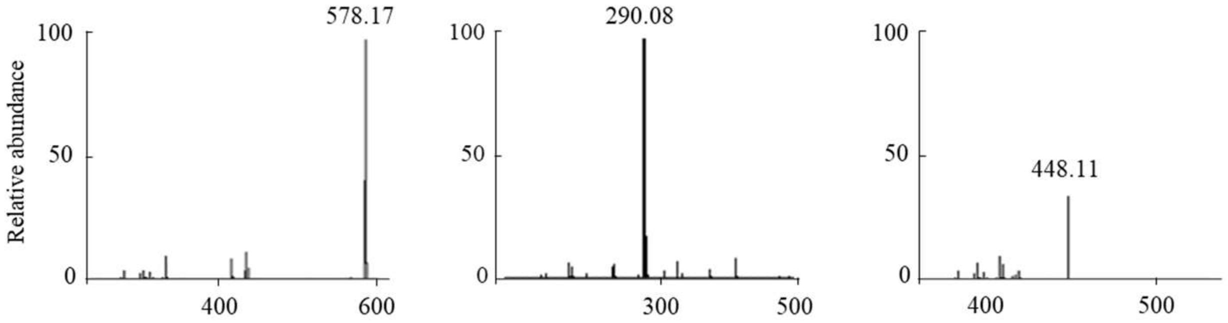

C

Compounds	RT (min)	MW (g/mol)	Mean ± SD (μg/mg)
Procyanidin B2	4.31	578.17	7.59 ± 0.06
Catechin	4.75	290.08	0.91 ± 0.05
Quercitrin	7.24	448.11	5.97 ± 0.09

Supplementary figure 2. Chemical constitutions and quantitative analysis of EFAX using ultrahigh-performance liquid chromatography-tandem mass spectrometry (UHPLC-MS/MS) chromatograms. EFAX and three reference standards were subjected to UHPLC analysis (A), and its three main compounds were detected in the high-resolution mass spectra: procyanidin B2 (m/z 578.17, $[M+H]^+$), catechin (m/z 290.08$[M+H]^+$) and quercitrin (m/z 448.11$[M+H]^+$) (B). The quantitative analysis of EFAX was conducted (C)
