# Supplementary material for: Ethyl Acetate Fraction of Amomum xanthioides Ameliorates Nonalcoholic Fatty Liver Disease in a High-Fat Diet Mouse Model
Source: Nutrients. 2020 Aug 13;12(8):2433. doi: 10.3390/nu12082433 (PMC7468949; doi:10.3390/nu12082433)
Supplement: Supplementary file 1 [file nutrients-12-02433-s001.zip › 20200721 Supplementary tables.docx]

| **Supplementary table 1.** Composition of normal chow diet and 60% high-fat diet | | | | | | | | | |
| --- | --- | --- | --- | --- | --- | --- | --- | --- | --- |
| Composition | |  | Normal diet | |  |  | High-fat diet | |  |
|  |  | g % | | kcal % | | g % | | kcal % | |
| Fat | | 4 | | 9 | | 34 | | 60 | |
|  | Carbohydrate | 72 | | 73 | | 26 | | 20 | |
|  | Protein | 18 | | 18 | | 26 | | 20 | |
|  | kcal/g | 4.0 | | | | 5.2 | | | |
